# Mathematical Model for an Effective Management of HIV Infection

**DOI:** 10.1155/2016/4217548

**Published:** 2016-02-28

**Authors:** Oladotun Matthew Ogunlaran, Suares Clovis Oukouomi Noutchie

**Affiliations:** ^1^Department of Mathematics and Statistics, Bowen University, Iwo 232101, Nigeria; ^2^MaSIM Focus Area, North-West University, Mmabatho 2735, South Africa

## Abstract

Human immunodeficiency virus infection destroys the body immune system, increases the risk of certain pathologies, damages body organs such as the brain, kidney, and heart, and causes death. Unfortunately, this infectious disease currently has no cure; however, there are effective retroviral drugs for improving the patients' health conditions but excessive use of these drugs is not without harmful side effects. This study presents a mathematical model with two control variables, where the uninfected CD4^+^T cells follow the logistic growth function and the incidence term is saturated with free virions. We use the efficacy of drug therapies to block the infection of new cells and prevent the production of new free virions. Our aim is to apply optimal control approach to maximize the concentration of uninfected CD4^+^T cells in the body by using minimum drug therapies. We establish the existence of an optimal control pair and use Pontryagin's principle to characterize the optimal levels of the two controls. The resulting optimality system is solved numerically to obtain the optimal control pair. Finally, we discuss the numerical simulation results which confirm the effectiveness of the model.

## 1. Introduction 

Acquired immunodeficiency syndrome (AIDS) is caused by a virus known as human immunodeficiency virus (HIV). Since HIV emerged in 1981, several studies, including mathematical modeling, have been devoted to understand the transmission of the infection. HIV models can be classified into two categories: population-level models and within-host models [1–9]. One of the major havocs wrought by the HIV is the destruction of CD4^+^T cells which play a significant role in the regulation of the body immune system. HIV causes a decline in the number of functional CD4^+^T cells thereby reducing the competency of the body defense mechanism to fight cell infections. Several mathematical models have been formulated to study the interactions between HIV and CD4^+^T cells [10–14]. Although HIV is not yet curable, there are antiretroviral drugs that help in boosting the immune system against cell infections. These antiretroviral drugs are categorized into two groups which are reverse transcriptase inhibitors (RTIs) and protease inhibitors (PIs). RTIs disrupt the conversion of RNA of the virus to DNA so that new HIV infection of cells is prevented. On the other hand, PIs hinder the production of the virus particles by the actively infected CD4^+^T cells [13].

In this paper, our objective is to present a within-host model which is a variant of the model proposed by Perelson and Nelson [7] with a saturated incidence. We incorporate two controls into the model and find the optimal treatment strategy that will produce maximum uninfected cells and minimum viral load with a minimum dose of drug therapies to prevent harmful effects associated with excessive use of drugs in the body.

## 2. Model Formulation

By assuming that the constant recruitment number of new uninfected cells and the number of death of uninfected cells have already been incorporated in the logistic growth function and that the rate of infection of CD4^+^T cells by free virions has been saturated probably because of overcrowding of free virions or as a result of protection measures being used by the HIV patient, and we obtain the variant model described by the following system of equations:

(1a) dTdt=rT1−TTmax−βVT1+αV,T0=T0≥0,

(1b) dIdt=βVT1+αV−μI,I0=I0≥0,

(1c) dVdt=NμI−γV,V0=V0≥0,

where *T* = *T*(*t*) denotes the concentration of uninfected CD4^+^T cells at time *t*, *I* = *I*(*t*) denotes the concentration of infected CD4^+^T cells, and *V* = *V*(*t*) is the concentration of free HIV at time *t*, *r* is the growth rate, *T*_max_ denotes the maximum CD4^+^T cells concentration in the body, *β* is the rate of infection of CD4^+^T cells by virus, and *α* is the saturation factor. *μ* is the per capita rate of disappearance of infected cells and *γ* is the loss rate of free virus. *Nμ* is the rate of production of virions by infected cells, where *N* is the average number of virus particles produced by an infected CD4^+^T cell. All parameters in the model are strictly positive.

Therefore, (1a) represents the rate of change of uninfected CD4^+^T cells with respect to time *t* in the HIV patient which is made up of the population of the uninfected cells minus the population of CD4^+^T cells which becomes infected in the process of time. Equation (1b) describes the rate of change of the HIV infected cells given as difference in the population of infected cells and the number of infected cells that disappear at time *t*. Lastly, the differential equation (1c) gives the rate of change of the population of the free HIV.

We now introduce a set of controls *u*(*t*) = (*u*_1_(*t*), *u*_2_(*t*)) into model (1a)–(1c) simulating the antiviral therapy. Then the model becomes

(2) dTdt=rT1−TTmax−1−u1βVT1+αV,T0=T0≥0,dIdt=1−u1βVT1+αV−μI,I0=I0≥0,dVdt=1−u2NμI−γV,V0=V0≥0.

The two control functions *u*_1_(*t*) and *u*_2_(*t*) are bounded Lebesgue integrable functions. The control *u*_1_(*t*) denotes the efficacy of drug therapy in blocking the infection of new cells, and the control *u*_2_(*t*) denotes the efficacy of drug therapy in inhibiting the production of virus. If, for instance, *u*_1_(*t*) = 1, the blockage is 100% effective. On the other hand, if *u*_1_(*t*) = 0, there is no blockage.

## 3. Optimal Control Problem

Typically, an optimal control problem has an objective functional *J*((*x*(*t*), *u*(*t*)), a set of state variables (*x*(*t*) ∈ *X*), and a set of control variables (*u*(*t*) ∈ *U*) in time *t*, 0 ≤ *t* ≤ *t*_*f*_.

In this study, we define our objective functional as

(3) $$\begin{array}{c} J\left(\;u_{1},u_{2}\;\right)=\overset{t_{f}}{\underset{0}{\int }}\left\{\;T\left(\;t\;\right)-\left(\;\frac{r_{1}}{2}u_{1}^{2}+\frac{r_{2}}{2}u_{2}^{2}\;\right)\;\right\}dt, \end{array}$$

where *r*_1_ and *r*_2_ are positive constants representing the relative weights attached to the drug therapies. Our goal is to seek to maximize the objective functional given by (3) by increasing the population of the uninfected CD4^+^T cells, reducing the viral load (the number of free virions), and minimizing the cost of treatment. In other words, we want to find an optimal control pair (*u*_1_^*∗*^(*t*), *u*_2_^*∗*^(*t*)) such that

(4) $$\begin{array}{c} J\left(\;u_{1}^{*}\left(\;t\;\right),u_{2}^{*}\left(\;t\;\right)\;\right)=\underset{\left(u_{1}^{*}\left(t\right),u_{2}^{*}\left(t\right)\right)\in U}{\max}J\left(\;u_{1}\left(\;t\;\right),u_{2}\left(\;t\;\right)\;\right), \end{array}$$

where *U* is the control set defined by
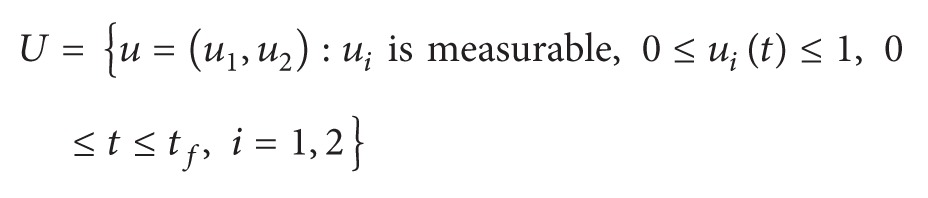
(5)

**Theorem 1 .**

Consider the control system (1a)–(1c). There exists an optimal control pair (*u*_1_^*∗*^(*t*), *u*_2_^*∗*^(*t*)) ∈ *U* such that

(6) $$\begin{array}{c} J\left(\;u_{1}^{*}\left(\;t\;\right),u_{2}^{*}\left(\;t\;\right)\;\right)=\underset{\left(u_{1}^{*}\left(t\right),u_{2}^{*}\left(t\right)\right)\in U}{\max}J\left(\;u_{1}\left(\;t\;\right),u_{2}\left(\;t\;\right)\;\right). \end{array}$$

**Proof**

See Appendix A.

Further, we discuss the necessary conditions that the optimal control must satisfy. We apply Pontryagin's maximum principle to the Hamiltonian function associated with system (2) which is given by

(7) Ht,u,T,I,V,λ1,λ2,λ3=LT,u,t+λ1dTtdt+λ2dItdt+λ3dVtdt,

where *L*(*T*, *u*, *t*) = *T* − (*r*_1_/2)*u*_1_^2^(*t*) + (*r*_2_/2)*u*_2_^2^(*t*) and *λ*_1_, *λ*_2_, and *λ*_3_ are adjoint functions to be determined appropriately.

**Theorem 2 .**

Let *T* ^*∗*^(*t*),  *I* ^*∗*^(*t*), and *V* ^*∗*^(*t*) be optimal state solutions with associated optimal controls *u*_1_^*∗*^, *u*_2_^*∗*^ for the optimal control problem (2) and (3). Then there exist adjoint variables *λ*_1_, *λ*_2_, and *λ*_3_ that satisfy the adjoint conditions:

(8) dλ1dt=−1−λ1r1−2TTmax−1−u1βV1+αIV−λ21−u1βV1+αV,dλ2dt=λ2μ−λ31−u2Nμ,dλ3dt=λ11−u1βT1+αV1−αV1+αV−λ21−u1βT1+αV1−αV1+αV+λ3γ,

with transversality conditions

(9) λ1tf=0,λ2tf=0,λ3tf=0.

In addition, the optimal control *u* ^*∗*^(*t*) is given by

(10) u1∗t=min⁡max⁡λ1t−λ2tβV∗T∗r11+αV∗,0,1,u2∗t=min⁡max⁡−NμI∗tλ2tr2,0,1.

**Proof**

See Appendix B.

By taking into consideration the property of the control space, the optimal control *u* ^*∗*^(*t*) is characterized as in (10). The optimal control pair and the state variables are determined by solving the following optimality system which consists of state system (2), adjoint system (8), and transversality conditions (9) together with the characterization of the optimal control pair (10):

(11) dT∗dt=rT∗1−T∗Tmax−1−u1∗βV∗T∗1+αV∗,dI∗dt=1−u1∗βV∗T∗1+αV∗−μI∗,dV∗dt=1−u2NμI∗−γV∗,dλ1dt=−1−λ1∗r1−2T∗Tmax−1−u1∗βV∗1+αV∗−λ21−u1∗βV∗1+αV∗,dλ2dt=λ2μ−λ31−u2∗Nμ,dλ3dt=λ11−u1∗βT∗1+αV∗1−αV∗1+αV∗−λ21−u1∗βT∗1+αV∗1−αV∗1+αV∗+λ3γ,T0=T0,I0=I0,V0=V0,λ1tf=0,λ2tf=0,λ3tf=0.

## 4. Numerical Simulations and Results

In order to solve the optimality system for the optimal control pair, we employ the Gauss-Seidel-like implicit finite-difference method known as the GSS1 method which was developed in 2001 by Gumel et al. [15]. For details about the method see [13, 15, 16]. By applying the method to approximate the state system forward in time and the adjoint system backward in time, we obtain

(12) Tk+1−Tkl=rTk+11−Tk+1Tmax−1−u1kβVkTk+11+αVk,Ik+1−Ikl=1−u1kβVkTk+11+αVk+1−μIk+1,Vk+1−Vkl=1−u2kNμIk+1−γVk+1λ1n−k−λ1n−k−1l=−1−λ1n−k−1r1−2Tk+1Tmax−1−u1kβVk+11+αVk+1−λ2n−k1−u1kβVk+11+αVk+1,λ2n−k−λ2n−k−1l=λ2n−k−1μ−λ3n−k1−u2kNμ,λ3n−k−λ3n−k−1l=λ1n−k−11−u1kβTk+11+αVk+11−αVk+11+αVk+1−λ2n−k−11−u1kβTk+11+αVk+11−αVk+11+αVk+1+λ3n−k−1γ.

Now using the following parameter and initial values

(13) r=0.03,β=0.000024,α=0.001,N=500,μ=0.02,γ=2.4,r1=200,r2=250,T0=1000,I0=400,V0=80,Tmax=1500,

and we performed numerical simulations for a period of 100 days to ascertain the effectiveness of the proposed model based on the disease progression before and after the introduction of treatment (a pair of controls). These parameter values are obtained from [3, 15, 17, 18].

**Figure 1:**
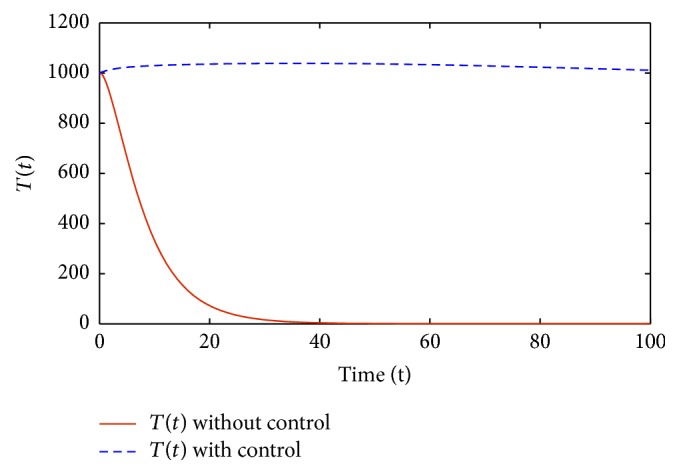
*T*(*t*) with and without control.

**Figure 2:**
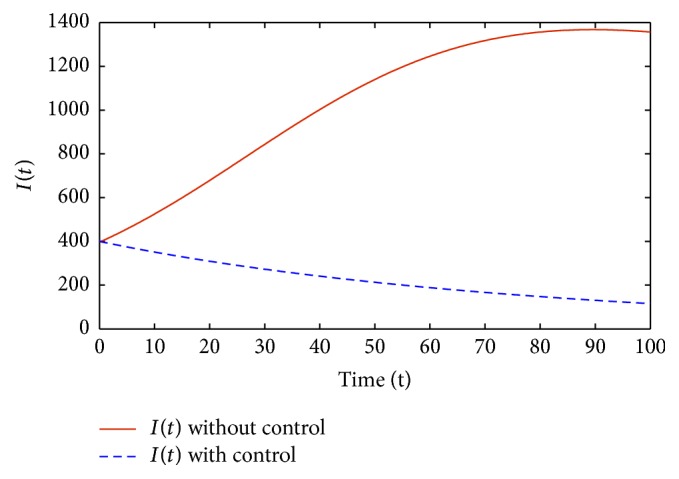
*I*(*t*) with and without control.

**Figure 3:**
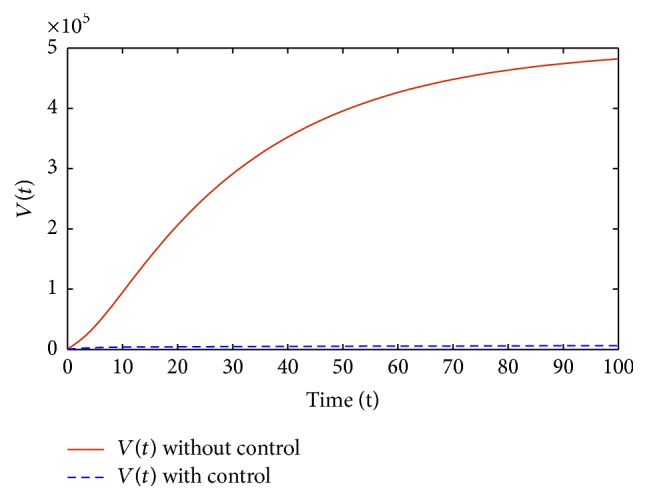
*V*(*t*) with and without control.

**Figure 4:**
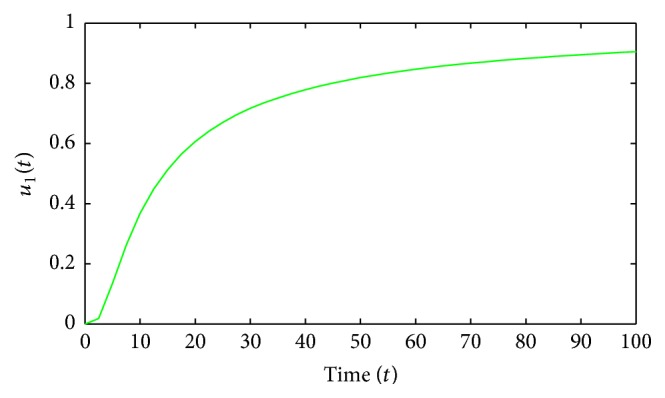
First optimal control.

**Figure 5:**
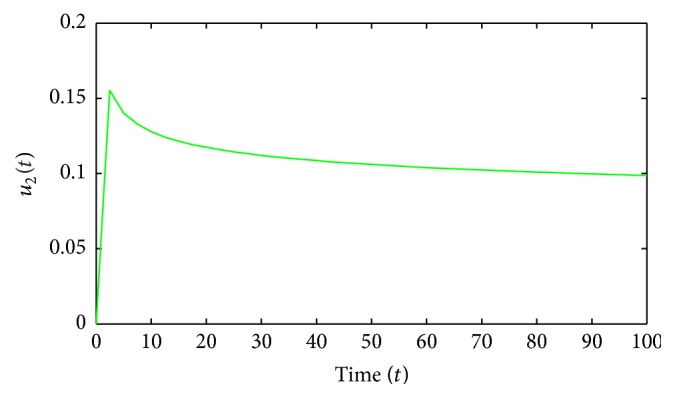
Second optimal control.

Figures 1–5 are the simulation results from which we can draw some conclusions on the effectiveness of drug therapies based on the concentrations of uninfected cells, infected cells, and free virus.  Figure 1 shows the population of uninfected CD4^+^T cells with and without control. Without treatments, the number of uninfected cells decreases drastically. On the other hand, with treatments the concentration of cells is maintained from the beginning to the end of the period.  Figure 2 shows the population of infected CD4^+^T cells with and without control. The concentration of infected cells decreases rapidly right from the very beginning of treatment and throughout the period of investigation; while the concentration of infected cells without treatment grows at the beginning and become stable toward the end of the period. Similarly in Figure 3, we see that the viral load increases drastically without treatments whereas with treatments there is no increase in the concentration of free virus. In fact, instead of the concentration to increase it reduces. In Figures 4 and 5, we see optimal treatments *u*_1_(*t*) and *u*_2_(*t*) required with the change in time to block new infection of cells and prevent viral production with minimum side effects.

## 5. Conclusion

In this paper, we have proposed and analyzed a mathematical model, with two control variables, describing HIV infection of CD4^+^T cells. The mathematical analysis of the proposed model, validated by the numerical simulation results shows the effectiveness of the model in maximizing the concentration of uninfected CD4^+^T cells, minimizing the concentrations of infected cells and free virions in the body with a minimum dose of combination of drug therapies in order to advert the adverse effects associated with excessive use of drug, and also indirectly minimizing the cost of treatment. Certainly, these results could be useful in developing improved treatment regimen towards addressing the challenge of HIV/AIDS.

## 6. Recommendation

Although there is presently no known cure for HIV/AIDS, there are now available antiretroviral HIV drugs which block infection of new cells and reduce viral load in the body and so HIV positive individual can now enjoy relatively good health and increased life expectancy. Early diagnosis with immediate commencement of the use of approved antiretroviral drugs before CD4^+^T cells levels fall below 350 cells/mm^3^, regardless of whether a person is showing signs of HIV or not, is highly advantageous. Most HIV/AIDS victims will have to take two or more drugs for the rest of their lives; however, antiretroviral HIV drugs also have side effects like any other drugs. In order to avoid or reduce these side effects, a rightful dose of an appropriate combination of these drugs is very essential. Therefore, it is important on the part of HIV positive individual to follow the antiretroviral treatment regimen. Governments especially in developing countries should be more responsive in providing improved health system and antiretroviral drugs for their teeming populations suffering and dying unnecessarily because they could not afford the drugs. Educational awareness programmes are still very much needed to prevent and contain the spread and for the proper management of the disease.

## Appendices

### A. Proof of Theorem 1

We use a result in Fleming and Rishel [19] and Hattaf and Yousfi [18] to prove the theorem. The optimal solution exists if the following hypotheses are satisfied:

(F1) The set of controls and corresponding state variables is nonempty.
(F2) The admissible control set *U* set is convex and closed.
(F3) The right hand side of the state system is bounded by a linear function in the state and control variables.
(F4) The integrand of the objective functional is concave on *U*.
(F5) There exists constants *k*_1_, *k*_2_ > 0 and *λ* > 1 such that the integrand *L*(*T*, *u*, *t*) of the objective functional satisfies
  (A.1) $$\begin{array}{c} L\left(\;T,u,t\;\right)\leq k_{2}-k_{1}{\left(\;{\left|\;u_{1}\;\right|}^{2}+{\left|\;u_{2}\;\right|}^{2}\;\right)}^{\lambda /2}. \end{array}$$

Obviously, condition (F1) is satisfied. By definition, control set *u*_1_, *u*_2_ ∈ *U* is convex and closed. State system (2) is bilinear in *u*_1_, *u*_2_, and so the right hand side of the system satisfies condition (F3), using the boundedness of the solutions. Also, the integrand of the objective functional is concave on control set *u*. Finally, we have the last requirement *L*(*T*, *u*, *t*) ≤ *k*_2_ − *k*_1_(|*u*_1_|^2^ + |*u*_2_|^2^)^*λ*/2^, where *k*_2_ depends on the upper bound on *T*, and *k*_1_ > 0 since *r*_1_, *r*_2_ > 0. Thus, there exists an optimal control pair.

### B. Proof of Theorem 2

To determine the adjoint equations and the transversality conditions, we use the Hamiltonian (7). By substituting *T*(*t*) = *T* ^*∗*^(*t*), *I*(*t*) = *I* ^*∗*^(*t*), and *V*(*t*) = *V* ^*∗*^(*t*) and differentiating the Hamiltonian with respect to *T*(*t*), *I*(*t*), and *V*(*t*), we obtain

(B.1) dλ1dt−∂H∂T=−1−λ1r1−2TTmax−1−u1βV1+αV−λ21−u1βV1+αV,dλ2dt−∂H∂I=λ2μ−λ31−u2Nμ,dλ3dt−∂H∂V=λ11−u1βT1+αV1−αV1+αV−λ21−u1βT1+αV1−αV1+αV+λ3γ.

Now, using the optimality conditions, we find

(B.2) $$\begin{array}{c} \frac{\partial H}{\partial u_{1}}=-r_{1}u_{1}+\frac{\lambda_{1}\beta V^{*}T^{*}}{1+\alpha V^{*}}-\frac{\lambda_{2}\beta V^{*}T^{*}}{1+\alpha V^{*}}=0. \end{array}$$

At *u*_1_ = *u*_1_^*∗*^(*t*), we have

(B.3) u1∗t=λ1−λ2βV∗T∗r11+αV∗,∂H∂u2=−r2u2−Nμλ3I∗t=0.

At *u*_2_ = *u*_2_^*∗*^(*t*), we find

(B.4) $$\begin{array}{c} u_{2}^{*}\left(\;t\;\right)=-\frac{N\mu \lambda_{3}I^{*}\left(t\right)}{r_{2}}. \end{array}$$
